# LL-37 but Not 25-Hydroxy-Vitamin D Serum Level Correlates with Healing of Venous Leg Ulcers

**DOI:** 10.1007/s00005-016-0423-9

**Published:** 2016-09-23

**Authors:** Alicja Krejner, Małgorzata Litwiniuk, Tomasz Grzela

**Affiliations:** 1 Laboratory of Cell Molecular Biology, Department of Histology and Embryology, Biostructure Research Centre, Medical University of Warsaw, Chalubinskiego 5, 02-004 Warsaw, Poland; 2 Postgraduate School of Molecular Medicine, Medical University of Warsaw, Warsaw, Poland; 3 Department of Otolaryngology, Medical University of Warsaw, Warsaw, Poland; 4 Clinic of Phlebology, Warsaw, Poland

**Keywords:** Cathelicidin, Healing rate, LL-37, Venous leg ulcer, Vitamin D

## Abstract

Human cathelicidin, LL-37, is small antimicrobial peptide, which reveals also some immunomodulatory and proangiogenic properties and, therefore, may promote wound healing. The expression of LL-37 is controlled by various factors, including vitamin D. Thus, any disturbances in vitamin D level may influence LL-37 production and, possibly, affect wound healing. Since deficiency of vitamin D was identified as a common problem in the population, this proof of concept study aimed to verify the relationship between serum levels of LL-37, vitamin D, and healing rate of venous leg ulcers. The study involved small group (*n* = 19) of patients with venous leg ulcers. Apart from non-venous ulcer aethiology, compression intolerance, active vein thrombosis, and wound infection, the exclusion criteria concerned also kidney insufficiency. The results of the analysis of wound healing rates were correlated with patients’ serum levels of 25(OH) vitamin D and LL-37. In addition, serum levels of pro-inflammatory cytokines (IL-6, IL-8, and TNF) were analyzed. We have found strong association between serum concentrations of LL-37 and the healing rates in patients with leg ulcers. Despite the fact that 25(OH) vitamin D levels in all patients were below the normal range, they did not show any correlation with healing rates. Furthermore, no association was observed between serum levels of 25(OH) vitamin D and LL-37. No significant correlation between tested pro-inflammatory cytokines and healing rate, LL-37, or 25(OH) vitamin D levels was also observed. Regardless of small study group, our results suggest that the assessment of serum concentration of LL-37, but not 25-hydroxy vitamin D, may help in predicting the wound healing efficacy. Moreover, this assessment may be useful in pre-selection of patients, which could benefit from local treatment with exogenous LL-37.

## Introduction

The standard treatment of venous leg ulcer comprises the use of compression therapy and proper local wound management. The compression allows the significant reduction of chronic venous hypertension in affected leg and thus improves the local blood perfusion (Partsch and Mortimer 2015). The local wound care, due to the use of various technologically advanced dressings, provides optimal conditions for tissue regeneration in the wound bed (Krejner and Grzela 2015; Waring and Parsons 2001). In majority of patients, this standard approach is sufficient enough to get good results. However, in some cases, despite the combination of effective compression and adequate local treatment, the wound healing is not satisfactory. Several detrimental factors were identified until now, including bacterial colonization of the wound, persisting chronic inflammation, or excess of destructive matrix metalloproteinases (Litwiniuk et al. 2009; Mustoe et al. 2006). However, delay in the wound healing may also be due to some other, so far superficially defined parameters, including genetic predisposition or metabolic state of the organism (Banerjee and Sen 2015; Shaw and Martin 2009; Weinstein et al. 2015). The last condition is highly sensitive to generalized malnutrition, or selective deficiency of particular agent. One of good examples of such agent could be vitamin D (Burkiewicz et al. 2012a, b; Zubair et al. 2013), especially since its deficiency was recently recognized as the common problem in the elderly population, who constitute the vast majority of patients with non-healing wounds (Houston et al. 2015).

Apart from its well known role in regulation of calcium-phosphate homeostasis, vitamin D was found to reveal some other activities, including regulation of immune response (Hewison 2012; Tiwari et al. 2014; Trochoutsou et al. 2015). One of postulated modes of vitamin D action in immunity could be regulation of expression of small cationic antimicrobial peptides, among them human cathelicidin (Doss et al. 2010; Gonzalez-Curiel et al. 2014; Grzela et al. 2012).

Human cathelicidin is synthesized by numerous cells as an inactive precursor, hCAP18/LL-37. It consists of a highly conserved N-terminal signal sequence, a conserved cathelin domain, and small antimicrobial C-terminal domain, known as LL-37 (Vandamme et al. 2012). Proteolytic cleavage of the hCAP18/LL-37 precursor leads to the release of antimicrobial LL-37 domain. This domain exhibits direct antimicrobial and anti-biofilm effect on a broad spectrum of Gram-positive and Gram-negative bacteria, viruses, and fungi (Barlow et al. 2011; Overhage et al. 2008; Wong et al. 2011). Besides its direct bactericidal activity, human cathelicidin can also reveal some indirect antimicrobial effects. It may attract various cells of the innate immune system to deal with pathogens; furthermore, it can influence the production and secretion of several pro- and anti-inflammatory cytokines. Thus, LL-37 may modify the inflammatory response (Agier et al. 2015; Wuerth and Hancock 2011). Interestingly, it was also recognized as regulator of angiogenesis and re-epithelialization (Heilborn et al. 2003; Koczulla et al. 2003). Therefore, due to its pleiotropic mode of action, LL-37 may be important for the healing of chronic wounds, including those infected with multiple, and biofilm-forming organisms (Ammons 2010).

The expression of hCAP18/LL-37 is controlled by various factors. Since the promoter region of the cathelicidin gene contains a vitamin D response element, also, the already mentioned vitamin D may play important role in that regulation (Gombart et al. 2005). It has been reported that in healthy adults, serum levels of LL-37 positively correlated with vitamin D concentrations (Dixon et al. 2012). On the other hand, vitamin D deficiency was suggested to impair the immune response (Gombart 2009). These observations may be of great clinical importance, due to previously mentioned common deficiency of vitamin D (Houston et al. 2015). Therefore, the aim of our proof of concept study was to verify the possible relationship between serum concentrations of vitamin D, human cathelicidin LL-37, and the healing of chronic venous leg ulcers. Considering the context of chronic inflammation associated with leg ulcers, to enable easier analysis of collected data, serum levels of pro-inflammatory cytokines [interleukin (IL)-6, IL-8, and tumor necrosis factor (TNF)] were also measured.

## Materials and Methods

The study involved 19 patients (12 female and 7 male and mean age 68.6 ± 13.8) with chronic venous leg ulcers present for at least 8 weeks, but not longer than 2 years. The venous insufficiency etiology of assessed wounds was confirmed by Duplex–Doppler ultrasound examination. The exclusion criteria involved clinical symptoms of wound infection within last 4 weeks preceding the assessment, arterial insufficiency with ankle-brachial pressure index <0.8 or >1.1, active deep vein thrombosis, clinically significant kidney insufficiency as well as any systemic disease in unstable stage. All patients received local wound management according to TIME (tissue-inflammation-moisture edges) strategy and recommendations of multidisciplinary expert group from Polish Wound Management Society (Jawień et al. 2011). The wound was covered by the hydrofiber-foam composite dressing (Aquacel Foam, ConvaTec, Deeside, UK), and multilayer compression was applied to the affected leg. All patients gave the informed consent to participate in the study that was approved by the local ethics committee and conducted according to ethical guidelines of the 1975 Declaration of Helsinki.

The wound healing rate was analyzed retrospectively, based on the results of routine measurements of wound area, performed within at least 1 month preceding the assessment. The measurements were done using Visitrak Digital device (Smith&Nephew, Largo, FL, USA) in weekly intervals. The results were used to calculate the percent reduction of an initial wound surface, expressed then as mean wound healing rate per week (Cardinal et al. 2008). To allow easier patients comparison, the mean healing rate below 5 % per week was arbitrarily considered as poor, 5–10 % (moderate), and 11–15 % (good), whereas the mean healing rate above 15 % per week was identified as fast.

The serum levels of 25-hydroxy vitamin D_3_, 25(OH)D_3_, were measured using the Vitamin D ELISA Kit (Cayman Chemicals, Ann Arbor, MI, USA) with the assay detection limit of 0.19 ng/ml. To reduce the influence of differential sun exposure on vitamin D level (Martineau et al. 2011), the study was conducted between October and March.

The levels of LL-37 in serum samples were determined using the Human LL-37 ELISA Kit (MyBioSource, San Diego, CA, USA). The test detection range was 1.56–100 ng/ml.

The cytokine levels in serum samples were measured in duplicates, using Human IL-6, IL-8, and TNF Ultrasensitive ELISA kits, respectively, according to detailed protocols provided by the manufacturer (all tests from Invitrogen, Camarillo, CA, USA). The absorbance of analyzed samples was estimated using the Microplate Reader 550 (BIO-RAD, Hercules, CA, USA). Based on the respective standard calibration curves, the OD results were converted to the specific cytokine concentrations (expressed in pg/ml). For all the tested cytokines, the assays sensitivity, corresponding to the lowest points of the standard calibration curves, was 0.4 pg/ml.

The data were calculated using the BrightStat online software (Stricker 2008).

## Results

The clinical characteristics of patients group were shortly summarized in Table 1.

**Table 1:** Summary of clinical data

Feature/parameter	Value
Age (years ± SD)	68.6 ± 13.8
Sex (female/male)	12/7
Venous insufficiency in:	–
Superficial system	19
Deep system	12
Mean wound size at the beginning of observation period (min/max)	59.6 cm^2^ (8.0–190.0)
Mean healing rate (% per week)	9.7 ± 6.6
Poor	3.5 ± 0.5
Moderate	6.8 ± 1.0
Good	13.0 ± 1.4
Fast	19.2 ± 2.2

**Fig. 1:**
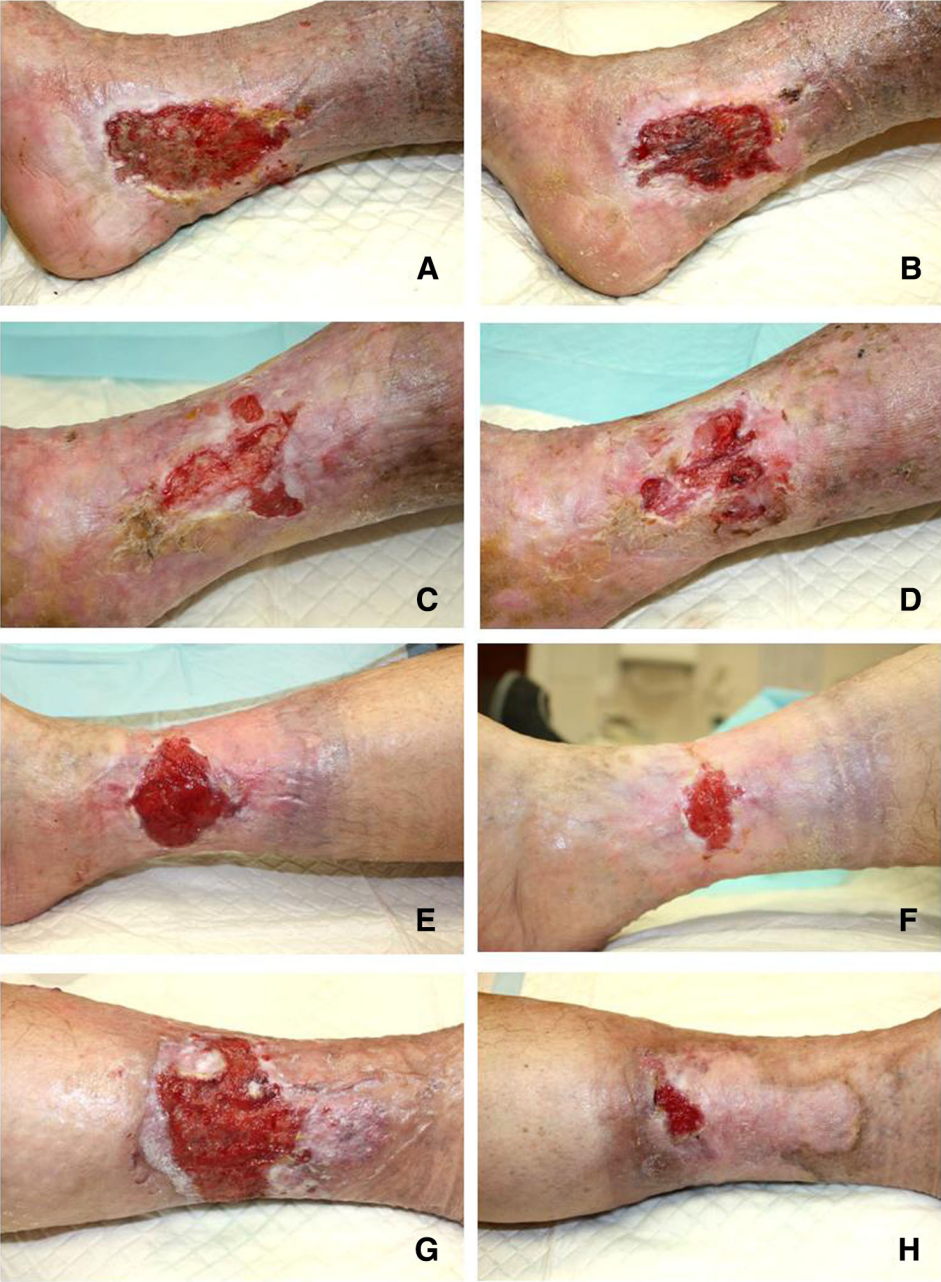
Examples of paired wound pictures representative for various healing patterns: **a**, **b** poor; **c**, **d** moderate; **e**, **f** good; and **g**, **h** fast healing. The pictures in *left column* (**a**, **c**, **e,** and **g**) show the wound at the beginning of observation period (day 0), whereas the pictures in *right column* (**b**, **d**, **f,** or **h**, respectively) show the same wounds after 4 weeks of standard treatment

The mean healing rate below 5 % per week, which was considered as poor, was observed in six patients, whereas the mean healing rate above 15 % per week, described as fast, was found in five individuals. The moderate and good healing rates were observed in six and two patients, respectively (Fig. 1).

**Fig. 2:**
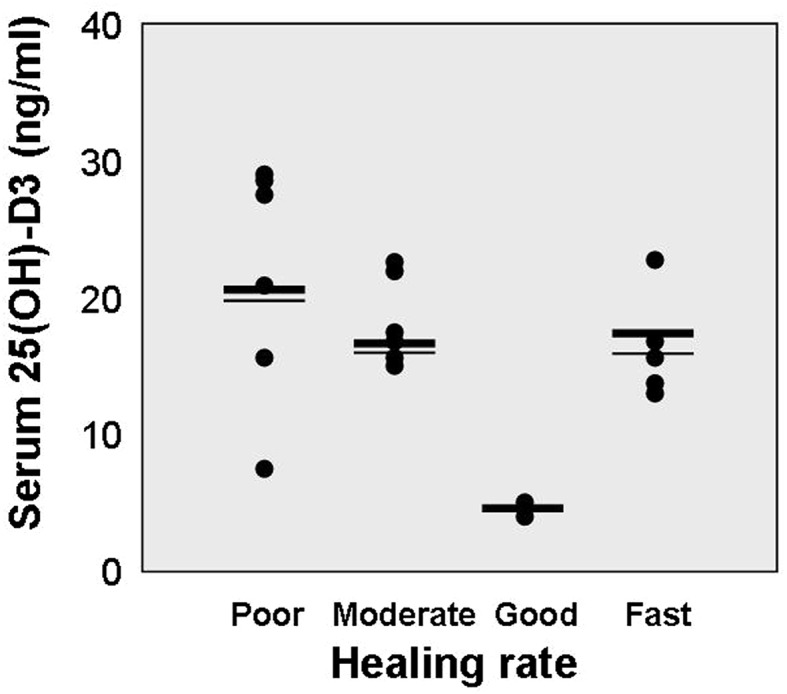
Serum concentration of 25-hydroxy-vitamin D (ng/ml) in relation to wound healing rates. Each *dot* represents data of one patient. *Bold lines* correspond to mean value, whereas *thin lines* represent median for respective groups

The concentrations of 25(OH)D_3_ in tested serum samples ranged from 4–29 ng/ml, with a mean value 18.2 ± 7.1 ng/ml. It did not show any correlation with the mean healing rate observed in analyzed patients (Fig. 2).

**Fig. 3:**
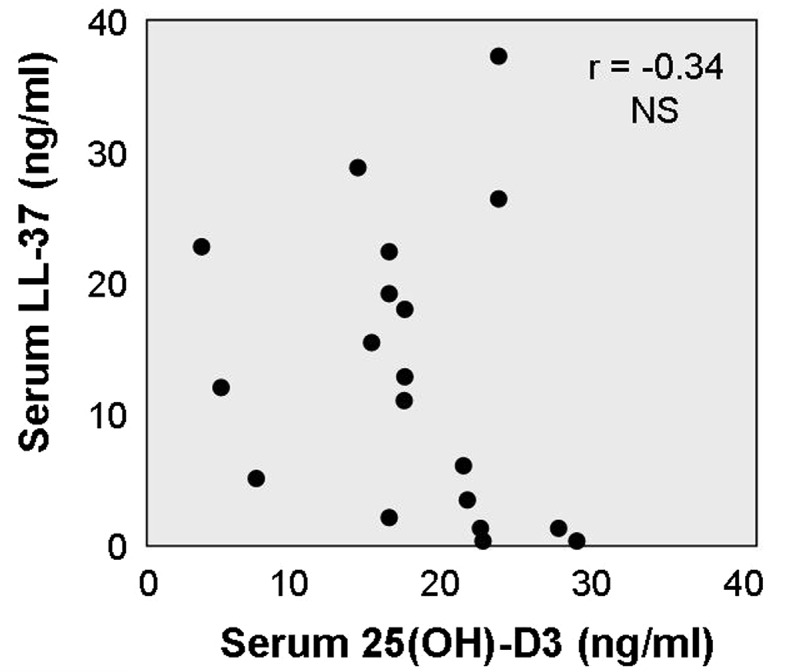
Association between serum levels of 25-hydroxy-vitamin D (ng/ml) and human LL-37 (ng/ml). Each *dot* represents data of one patient. *NS* non-significant

**Fig. 4:**
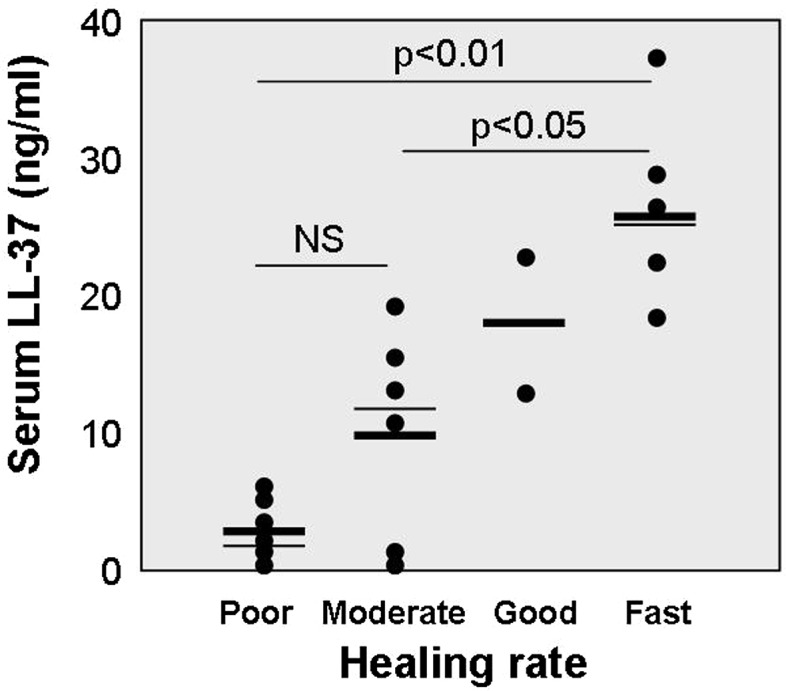
Serum concentration of LL-37 (ng/ml) in relation to wound healing rates. Each *dot* represents the data of one patient. *Bold lines* correspond to mean values, whereas *thin lines* represent median for respective groups. Statistical significance was calculated using Mann–Whitney *U* test

The mean serum level of LL-37 was 12.2 ± 11.1 ng/ml, with range 0.1–37.6 ng/ml. Interestingly, serum levels of LL-37 did not reveal statistically significant correlation with serum concentrations of 25(OH)D_3_ metabolite (Fig. 3). On the other hand, serum concentrations of LL-37 were strongly associated with calculated mean healing rates. Thus, the lowest serum levels of LL-37 were found in patients with poor (mean 2.6 ± 2.2 ng/ml) and moderate wound healing (mean 9.8 ± 7.6 ng/ml), whereas higher values of LL-37 were observed in patients with good and fast healing rates (mean 17.7 ± 7.3 ng/ml and 26.6 ± 7.2 ng/ml, respectively) (Fig. 4).

The mean serum levels of tested pro-inflammatory cytokines were: 3.5 ± 2.9 pg/ml for IL-6, 6.3 ± 5.6 pg/ml for IL-8, and 10.6 ± 2.5 pg/ml for TNF. Although slight associations were observed between all the tested cytokines, as well as between IL-6 or IL-8 and 25(OH) vitamin D metabolite, none of observed trends reached statistical significance.

## Discussion

The results of our proof of concept study support other reports showing that vitamin D deficiency is the common issue, particularly in elderly population. Noteworthy, when considering 30 ng/ml of 25(OH)D_3_ as the lowest sufficient serum level (Holick et al. 2011), none of tested individuals from our study group reached normal value. This observation may be clinically relevant, especially in context of widely discussed various non-skeletal activities of vitamin D, including the modulation of immune response or tissue regeneration (Gunville et al. 2013; Hewison 2012; López-López et al. 2014; McKenna et al. 2015; Ross et al. 2011; Trochoutsou et al. 2015). However, in our vitamin D-deficient patients, we did not observe any association between serum levels of 25(OH)D_3_ and wound healing rates. The lack of such association may suggest that the postulated role of vitamin D in chronic wound healing, if any, is rather indirect and should be considered as a component of more complex mechanisms.

One of hypothetical mechanisms may concern previously mentioned the involvement of vitamin D in the regulation of promoter activity and the expression of human cathelicidin LL-37 (Dixon et al. 2012). However, we did not find any correlation between serum levels of 25(OH)D_3_ and LL-37. Interestingly, similar lack of correlation between serum levels of 25(OH)D_3_ and LL-37 was also observed in another study, in patients with active tuberculosis (Yamshchikov et al. 2010). Possibly, such discrepancy may be due to different metabolites of vitamin D, analyzed in mentioned studies. The authors of previously mentioned report have tested 1,25(OH)D_3_, whereas we have assessed serum levels of 25(OH)D_3_. On the other hand, although 1,25-hydroxy D_3_ represents active form of this vitamin, in patients without clinically overt kidney insufficiency, the level of 25(OH)D_3_ may also be used as the good indicator of vitamin D deficiency (Holick et al. 2011; Ross et al. 2011). One has to keep in mind that the lack of association may also result from small number of patients, too small, to find any statistically significant correlation. However, even despite very small group, we have observed considerable association between serum concentrations of LL-37 and mean wound healing rates. The observed difference did not correlate with serum levels of selected pro-inflammatory cytokines (IL-6, IL-8, or TNF). Moreover, those levels did not differ among patients groups, thus suggesting that, possibly, at least in our observation, LL-37 levels and healing rates may represent rather individual patient properties, than actual inflammatory status of the wound.

Noticeably, the serum concentrations of LL-37 may differ from those in wound fluid, e.g., as the result of its extensive local production (Heilborn et al. 2010). Therefore, if available, exudates should obviously be considered as the better material for studies focused on the wound healing. Regrettably, exudates collection in some patients, especially with small wounds, or with less exudate amounts, may be impossible, or at least very difficult (Cutting 2003). On the other hand, the overall concentration of biologically active factors in the serum strongly determines the composition of wound exudates (Forsberg et al. 2008). To verify, whether serum levels of LL-37 correspond to those in wound exudates, we have compared both the types of samples in eight patients from our small study group. We have found that the concentrations of LL-37 in all the tested exudates were even up to tenfold higher, as compared to respective serum samples. However, even despite different nominal values, the LL-37 levels in wound exudates reflected those observed in serum samples. Hence, low serum levels corresponded to lower concentration in exudate samples, whereas high levels of LL-37 in exudates were observed in patients with its higher concentrations in serum (data not shown). Noteworthy, as already mentioned, high levels of LL-37 in serum and/or wound fluid were associated with faster wound healing. On the other hand, low concentrations of LL-37 in serum (and, presumably, in wound exudates) correlated with poor and slow healing rate.

When considering postulated pleiotropic action of LL-37 in pathophysiology of chronic wounds (Dressel et al. 2010; Heilborn et al. 2003), the assessment of endogenous LL-37 concentration in serum or, if available, in wound exudates, may be useful to predict the expected healing efficacy. On the other hand, our data provide the rationale for use of exogenous LL-37 in treatment of chronic wounds. Indeed, recently, Grönberg et al. (2014) have shown that the topical application of 0.5 or 1.6 mg/ml of human recombinant LL-37 significantly accelerated reduction of wound surface in patients with venous leg ulcer. Interestingly, the authors of that study have found that the best clinical effect was limited to low and mid doses of LL-37, whereas a dose of 3.2 mg/ml appeared to be less effective. Moreover, such a high dose was associated with increased risk of adverse reaction (ulcer necrosis, or severe inflammatory reaction). Possibly, the measurement of serum LL-37 levels would enable the selection of patients, who especially benefit from local application of this factor. According to that we hypothesize, that in patients with high endogenous levels of LL-37, we could expect good healing efficacy using the standard therapy only, whereas individuals with low endogenous LL-37 could be good candidates for topical wound treatment with recombinant LL-37. Nevertheless, due to small patients group, this issue still requires further studies.
